# Bonds broken and formed during the mixed-linkage glucan : xyloglucan endotransglucosylase reaction catalysed by *Equisetum* hetero-trans-β-glucanase

**DOI:** 10.1042/BCJ20160935

**Published:** 2017-03-08

**Authors:** Thomas J. Simmons, Stephen C. Fry

**Affiliations:** The Edinburgh Cell Wall Group, Institute of Molecular Plant Sciences, The University of Edinburgh, The King's Buildings, Edinburgh EH9 3BF, U.K.

**Keywords:** cell walls, *Equisetum*, hetero-transglycosylation, mixed-linkage glucan, transglycanases, xyloglucan

## Abstract

Mixed-linkage glucan∶xyloglucan endotransglucosylase (MXE) is one of the three activities of the recently characterised hetero-trans-β-glucanase (HTG), which among land plants is known only from *Equisetum* species. The biochemical details of the MXE reaction were incompletely understood — details that would promote understanding of MXE's role *in vivo* and enable its full technological exploitation. We investigated HTG's site of attack on one of its donor substrates, mixed-linkage (1→3),(1→4)-β-d-glucan (MLG), with radioactive oligosaccharides of xyloglucan as the acceptor substrate. Comparing three different MLG preparations, we showed that the enzyme favours those with a high content of cellotetraose blocks. The reaction products were analysed by enzymic digestion, thin-layer chromatography (TLC), high-pressure liquid chromatography (HPLC) and gel-permeation chromatography (GPC). *Equisetum* HTG consistently cleaved the MLG at the third consecutive β-(1→4)-bond following (towards the reducing terminus) a β-(1→3)-bond. It then formed a β-(1→4)-bond between the MLG and the non-reducing terminal glucose residue of the xyloglucan oligosaccharide, consistent with its xyloglucan endotransglucosylase/hydrolase subfamily membership. Using size-homogeneous barley MLG as the donor substrate, we showed that HTG does not favour any particular region of the MLG chain relative to the polysaccharide's reducing and non-reducing termini; rather, it selects its target cellotetraosyl unit stochastically along the MLG molecule. This work improves our understanding of how enzymes can exhibit promiscuous substrate specificities and provides the foundations to explore strategies for engineering novel substrate specificities into transglycanases.

## Introduction

The cell wall, which surrounds virtually all plant cells, has a skeleton of cellulosic microfibrils tethered (and also separated) by a hydrated extraprotoplasmic matrix composed mainly of carbohydrates. Cell expansion is regulated by cell-wall extensibility, which itself is controlled in part by modification of cell-wall polymers by apoplastic enzymes. In addition, the cell wall confers strength to mature tissues, pest and pathogen defence [1], cell signalling and cell–cell adhesion; the wall also determines cell morphology [2] and serves as a source of biologically active signalling molecules [3,4]. Owing to the presence of enzymes that target the structural polysaccharides, plant cell walls are dynamic structures.

The primary cell wall is typically construed as being composed largely of three structurally and functionally distinct polysaccharide fractions: cellulose, hemicelluloses and pectins. The occurrence, prevalence and structure of different cell-wall components often exhibit phylogenic, cell-type and stimulus-dependent variation, the result of the different evolutionary pressures and the differing characteristics conferred on cell walls by each component.

The hemicellulose xyloglucan comprises a (1→4)-linked β-d-glucopyranosyl backbone with various substitution patterns. Typically, the first two or three of every four consecutive glucose residues are α-d-xylosylated at position 6 (hence XXGG, XXXG [5]; see Fry et al. [6] for a description of xyloglucan nomenclature and Franková and Fry [7] for an update). These xyloglucan ‘base units’ are then further decorated by other residues, providing a wide scope for final xyloglucan structures. Xyloglucan side chains can also vary widely in different plant tissues and species [5]. The phylogenic distribution of xyloglucan is a case in point for the significance of changes in cell-wall content to plant evolution and adaptation. Xyloglucan chains avidly hydrogen-bond to cellulosic surfaces and are proposed to tether adjacent microfibrils [8], possibly through local xyloglucan/cellulose nodes [9]. Xyloglucan appears ubiquitous in the embryophytes and absent or minor in algae [10–12], suggesting a pivotal role in the transition from water to the colonisation of land.

Mixed-linkage (1→3),(1→4)-β-d-glucan (MLG) — another hemicellulose — is an unbranched β-d-glucopyranosyl homopolysaccharide in which cello-oligosaccharide regions [(1→4)-linked stretches of variable length, but typically 3–4 residues] are connected by single (1→3) bonds; these (1→3) bonds are never found consecutively [13,14]. Owing to their structural similarity to cellulose, the (1→4)-bonded stretches are commonly referred to as cellotriosyl and cellotetraosyl units. These units are relatively rigid, while the (1→3) bonds interconnecting them serve as hinges, giving the MLG molecule a kinked appearance, with overall flexibility [15]. Like xyloglucan, MLG can hydrogen-bond to cellulose and may serve a role in tethering adjacent microfibrils [8].

In contrast with the wide phylogenic distribution of xyloglucan among land plants, MLG is known only in two widely separated plant lineages: the Poales (grasses, cereals, reeds and their relatives) [11,16] and *Equisetum* (horsetails, an evolutionarily isolated genus of non-flowering vascular plants) [17–19]. The phylogenetic distance between the Poales and *Equisetum* indicates that the acquisition of MLG is most probably an example of convergent evolution. MLG also occurs in some lichens, e.g. *Cetraria islandica* (Iceland ‘moss’, whose MLG is known as lichenan) [20]. MLG has not been biochemically detected in the majority of plants, algae or fungi, although there is immunological evidence for an MLG-like epitope in various ferns [21].

Ferns form a monophyletic group that is the sister clade to seed plants [22]. Within the ‘ferns’ (*sensu lato*), *Equisetum* is the earliest diverging extant group [23]; indeed, *Equisetum* is probably the most evolutionarily isolated of all extant land-plant genera, and its ancestors are thought to have diverged from its closest living relatives >370 Mya [24,25]. Fittingly, in view of the evolutionary isolation of *Equisetum* and the distinctiveness of its general morphology, *Equisetum* cell walls are also unique, being composed of a distinct complement of polymers that transcend the typical type I∶type II cell-wall classification. While containing high levels of MLG — characteristic of type II cell walls — they lack type II levels of (glucurono)xylans and contain high levels of pectin — features characteristic of type I walls [11,17,18,26].

In both Poales and *Equisetum*, the presence of MLG may be related to their shared high silica content [17,27,28]. However, there is evidence to suggest that MLG can play different roles in these distinct taxa. For example, in the Poales, MLG is often metabolically labile, being hydrolysed to glucose shortly after germination and thus serving as a carbohydrate reserve [29,30]; there is no evidence for this in *Equisetum*, whose MLG tends to be abundant in both young and senescing tissues [18]. Furthermore, the presence of MLG∶xyloglucan endotransglucosylase activity (MXE; see below) in *Equisetum*, but not in members of the Poaceae [31,32], suggests another distinct function.

Plants make use of many different mechanisms to modulate the extensibility of their cell walls [33], a commonly proposed mechanism being the covalent modification of hemicelluloses by apoplastic transglycanases [31,34–39]. Transglycanases catalyse the endo-cleavage of a donor polysaccharide followed by the formation of a glycosidic bond between the new potentially reducing terminus and the non-reducing terminus of another oligo- or poly-saccharide (the acceptor substrate). While transglycanases are thought *in vivo* to catalyse endotransglycosylation between polysaccharides, *in-vitro* assays are typically performed using a polysaccharide as the donor and a labelled oligosaccharide as the acceptor. There is, however, evidence that polysaccharide-to-polysaccharide endotransglycosylation does occur [40,41].

The best known cell-wall transglycanase activity is xyloglucan endotransglucosylase (XET) — a homo-transglycanase which uses xyloglucan as both the donor and the acceptor substrate [34,35]. XET activity is exhibited by members of the CAZy glycosylhydrolase family 16 (GH16) and appears ubiquitous in the land plants. It is proposed to play crucial roles in cell-wall physiology. As well as containing XET-active enzymes, family GH16 also contains xyloglucan endohydrolase (XEH)-active enzymes. In acknowledgement of the biochemical similarity of the XET and XEH reactions, and because the structural (primary and tertiary) similarity of the GH16 enzymes exhibiting them suggests a distinct phylogenetic clade, the nomenclature recognises xyloglucan endotransglucosylase/hydrolase (XTH) as a subfamily that encompasses all XET- and XEH-active GH16 enzymes of plants [42,43].

Despite over 20 years' study, the precise roles of XTHs remain conjectural. A frequently observed negative correlation between extractable XET activity and cell age led to the suggestion that XET may be performing the hypothesised role of catalysing endotransglycosylation in controlled wall-loosening during cell expansion [34]; it was also shown that XET activity participates in cell-wall assembly [41]. More recent reports have further implicated XET activity in regulated cell expansion [44–48].

XTHs have also been implicated in other physiological phenomena, such as cell-wall restructuring [41], the development of vascular tissues [49] and, by allowing the integration of newly secreted xyloglucans into the pre-existing cell-wall architecture, cell-wall assembly [50]. Furthermore, it appears that some XTHs may also, in some situations, be involved in the cessation of cell expansion. Mellerowicz et al. [51] suggest that XTH proteins produced during wood secondary cell-wall development are involved in the strengthening of xylem tissues. Maris et al. [52] showed that application of *At*XTH14 or *At*XTH26 to onion epidermal peels during constant-load extensiometry actually decreased wall extensibility. Some evidence supports the hypothesis that XTHs may exhibit functional disparity in response to varying conditions. Nishikubo et al. [53] proposed that the ratio of newly synthesised xyloglucan to XTH within Golgi vesicles can dictate whether XET action strengthens or loosens the cell wall. Vissenberg et al. [54] showed that the localisation of XET action can vary with the mechanical properties of the cell wall.

Preferences in site of attack on the donor substrate could have significant ramifications for the roles of transglycanases. XTHs have been reported to exhibit distinct preferences with regard to the length of their substrates. For example, *Vinca* XTH1 only acted efficiently when using donor xyloglucans of over 10 kDa [35]. Tabuchi et al. [55] characterised an XTH from azuki bean epicotyls, which transferred 50-kDa portions from high-*M*_r_ xyloglucans to labelled xyloglucan-derived oligosaccharides (XGOs) and separately hydrolysed the high-*M*_r_ xyloglucans to 50-kDa products; xyloglucans of 60 kDa were not hydrolysed at all. In contrast, other work [56–60] has shown that (for some XTHs) large oligosaccharides can function as donor substrates, albeit at a lower rate than with polysaccharides. With regard to acceptor substrate, *Vigna* XTH1 acted equally regardless of differences in size [35], whereas recombinant *Arabidopsis* XTH22 protein had a much higher affinity for xyloglucan polysaccharides (*K*_m_ = 0.3 µM) than for the nonasaccharide XLLGol [40] (*K*_m_ = 73 µM). It remains unclear how XTHs might be able to ‘measure’ glycans of far greater size than them. Such abilities to measure xyloglucan length may have dramatic effects on XET roles *in situ*. Nonetheless, it appears that most XTHs exhibit a stochastic cleavage profile [61].

A kingdom-wide screen for novel transglycanases led to the surprise finding of the first predominantly hetero-transglycanase in extracts of all *Equisetum* species tested. This activity, MXE, catalyses a reaction identical with that of XET activity, but uses MLG as the donor substrate instead of xyloglucan [31]. Among land plants, both appreciable extractable MXE activity [31] and *in-vivo* MXE action [32] appear to be confined to *Equisetum*. A correlation of extractable MXE activity with organ age led Fry et al. [31] to suggest that MXE might function in the strengthening of ageing stems. Further studies showed MXE to be the most prevalent of three activities [MXE, XET and cellulose:xyloglucan endotransglucosylase (CXE)] that are exhibited by an *Equisetum* XTH subfamily member, termed hetero-trans-β-glucanase (HTG) [62]. The enzyme's preferred donor substrates are MLG and cellulose; thus, its MXE and CXE activities exceed its XET activity. By far the preferred acceptor substrates are xyloglucan-related oligosaccharides (and thus presumably also xyloglucan); HTG cannot appreciably utilise oligosaccharides of cellulose, MLG, xylan or mannan as acceptor substrates [62].

Here, by using a variety of biochemical approaches, we provide the first detailed characterisation of the MXE reaction. Since all non-*Equisetum* land plants tested essentially lack detectable MXE activity and action [32], and since our study of the *Equisetum fluviatile* transcriptome showed only a single HTG sequence [62], we can assume that when *Equisetum* extracts are tested for MXE activity, only a single protein is responsible. Using the native *Equisetum* enzyme also has an advantage over using the enzyme heterologously produced in *Pichia* [62] as we can guarantee correct glycosylation and any other post-translational modifications. We have characterised the site of attack and mechanism of recognition of MLG by HTG extracted from *Equisetum*. We identify HTG's site of attack on MLG as a β-(1→4)-bond situated exactly 3 residues following a β-(1→3) bond, though it shows little preference for the site of attack along the whole polymer chain relative to its reducing and non-reducing termini. We further present evidence that HTG forms new β-(1→4) bonds, as is consistent with its XTH subfamily membership. This work improves our understanding of how enzymes can exhibit promiscuous substrate specificity and provides the theoretical foundations to explore strategies for engineering novel substrate specificities into glycanases and for exploring the technological exploitation of MXE activity on different lignocelluloses.

## Materials and methods

### Materials

Microbial XEH [63] was a generous gift from Novo Nordisk A/S, Bagsværd, Denmark. Lichenase (from *Bacillus subtilis*; 330 U mg^−1^), β-d-glucosidase (from *Aspergillus niger*; 52 U mg^–1^), cellobiohydrolase [CBH1, from *Trichoderma longibrachiatum*; 0.07 U mg^–1^; possessing <1% endo-β-(1→3)-d-glucanase activity], cellulase (EGII, from *T. longibrachiatum*), cellulase (from *A. niger*), barley MLG (medium viscosity) and Iceland moss MLG (lichenan) were from Megazyme, Inc., Bray, Ireland. Merck (0.2 mm) silica-gel 60 TLC plates were from VWR (Lutterworth, U.K.). Tamarind xyloglucan was a generous gift from Dr K. Yamatoya (Dainippon Pharmaceutical Co., Osaka, Japan). *Equisetum arvense* MLG was purified as described previously [64]. Reductively tritiated XGOs ([^3^H]XGO-ols) were synthesised in-house, essentially as described by Hetherington and Fry [65], with XXXG from Megazyme and XXXGXXXG obtained by partial XEH digestion of tamarind xyloglucan. The specific radioactivities of [^3^H]XXXGol and [^3^H]XXXGXXXGol were ∼100 and 1.6 MBq mol^–1^, respectively. Dialysis tubing (12–14-kDa cut-off) was from Medicell International, Ltd, London, U.K. Miracloth was from Calbiochem (http://www.emdbiosciences.com). Solvents were from Fisher Scientific, U.K. Other chemicals came from Sigma–Aldrich, U.K.

### Preparation of size-homogeneous barley MLG

Three 10-ml aliquots of barley MLG (6.25 mg/ml) were separately fractionated by GPC on Sepharose CL-6B (bed volume 250 ml, internal diameter 1.4 cm, from which ∼3.6-ml fractions were collected) and the fractions with *K*_av_ [elution volume on GPC relative to totally excluded polymers (*K*_av_ = 0.0) and totally included low-*M*_r_ material (^3^H_2_O; *K*_av_ = 1.0)] 0.2–0.3 were pooled. The pool was rerun through the same column, and again the *K*_av_ 0.2–0.3 region was pooled as size-homogeneous MLG; the final yield was 7.7% of the total starting MLG.

### Plant material

*E. fluviatile* was grown outdoors in the King's Buildings pond in Edinburgh.

### Enzymic hydrolysis methods

For XEH digestion, 0.001% (w/v) microbial XEH in buffer [pyridine:acetic acid:water (1:1:98 by vol.), pH 4.7, containing 0.5% (w/v) chlorobutanol] was incubated with the sample at room temperature for 1 h.

For lichenase digests, lichenase was dissolved at 2 U ml^−1^ in the same buffer. One volume of lichenase solution was added to 1 volume of substrate (MLG) solution and incubated at room temperature for 4 h.

For glucosidase digestion, the sample was incubated with β-d-glucosidase (0.125 U ml^–1^ in the same buffer) at 20°C for various times.

All enzyme reactions were stopped by the addition of 0.5 volumes of 50% (v/v) formic acid.

### MXE reaction

To investigate the MXE reaction, we incubated *E. fluviatile* extracts, prepared in buffer as before (Fry et al. [31]), with a [^3^H]XGO-ol (acceptor substrate) and MLG (donor substrate). Routinely, the reactions were stopped by the addition of 0.5 volumes of 50% (v/v) formic acid. MXE products (MLG–[^3^H]XGO-ol) were freed from unreacted oligosaccharides by precipitation with ethanol (75%, v/v; overnight at 20°C). After bench-centrifugation, the pellet was redissolved in water at 100°C and then reprecipitated with 75% ethanol. This was repeated until the ethanolic supernatant contained no detectable radioactivity (typically four times). Details of the MXE reaction conditions for specific experiments are given in the following three paragraphs.

- For determination of the nature of the bond formed between MLG and xyloglucan (reported in Figure 3), a reaction mixture (1 ml) containing 15 kBq of reductively tritiated xyloglucan tetradecasaccharide ([^3^H]XXXGXXXGol), 0.75% (w/v) barley MLG and 50% (v/v) *E. fluviatile* extract was incubated at 25°C for 72 h, after which 46% of radioactivity had been incorporated into 75% (v/v) ethanol-insoluble material (MLG–[^3^H]XGO).
- For identification of the site of MXE cleavage of various MLGs (reported in Figure 4), the MXE products (MLG–[^3^H]XGO) were formed by incubation of reaction mixtures (20 µl) containing 15, 10 and 5 kBq [^3^H]XXXGol (84 MBq µmol^−1^) with 0.3% (w/v) *E. arvense*, barley and Iceland moss MLG, respectively, and 50% (v/v) *E. fluviatile* enzyme extract, for 16 h at 25°C. Ethanol was then added to 75% (v/v), which precipitated the MLG–[^3^H]XGO.
- For exploration of the preferred regions of MLG cleavage during the MXE reaction (reported in Figure 6), we incubated a 250-µl reaction mixture containing size-homogeneous MLG (8 mg/ml), [^3^H]XXXGol (1.5 µM; 37.5 kBq/250 µl) and a 40%-saturated (NH_4_)_2_SO_4_-precipitated enzyme preparation from *E. fluviatile* at 20°C. After 0, 2, 4 or 8 h, a 62.5-µl aliquot was stopped with formic acid, and the products were size-fractionated by GPC on Sepharose CL-6B.

### Chromatography

TLC was performed on 20 × 20-cm silica-gel plates pre-washed in acetone/acetic acid/water (1:1:1 by vol.). The chromatography solvent was butan-1-ol/acetic acid/water (BAW, 2:1:1), with one or two ∼8-h ascents. A malto-oligosaccharide ladder plus glucose was used as a marker mixture. Sugars were stained with thymol/H_2_SO_4_ [66].

GPC was performed on Bio-Gel P-2 (bed volume 166 cm^3^ and internal diameter 1.5 cm), in pyridine/acetic acid/water (1:1:98 by vol.) or on Sepharose CL-6B (bed volume 250 ml and internal diameter 14 mm) in pyridine/acetic acid/water (1:1:23 by vol.). Columns were calibrated with ^3^H_2_O (*K*_av_ = 1) and 40-MDa dextran (*K*_av_ = 0).

HPLC (specifically, ‘high-performance anion-exchange chromatography’; HPAEC) was performed on a CarboPac PA1 column (Dionex, Camberley, U.K.) with elution at 1 ml min^−1^. The eluent profile was 0.1 M NaOH, which also contained 0–30 min, 0–0.3 M sodium acetate (linear gradient); 30–36 min, 1 M sodium acetate; 36–42 min, 0 M sodium acetate. A pulsed amperometric detector with a gold electrode was used. For preparative HPLC, collected fractions were slightly acidified with acetic acid (to pH <6) immediately, then desalted by cation-exchange on Dowex-5 (H^+^ form; Sigma–Aldrich).

### Hexose assay

Two volumes of 0.2% (w/v) anthrone in concentrated sulphuric acid were added to one volume of sample (aqueous solution containing <0.1 mg/ml hexose) and the mixtures were thoroughly vortexed before incubation at 100°C for 5 min. Samples were cooled in an ice bucket before being measured for absorbance at 620 nm.

### Detection of radioactivity

Radioactive spots on TLC plates were localised by fluorography [66a] on pre-flashed Kodak BioMax MR film. Radioactivity in solution was assayed by scintillation counting in ScintiSafe3 (Fisher Scientific UK, Loughborough).

## Results

### Defining the structure of the MXE product

Since β-(1→3) bonds act as ‘hinges’ between the relatively rigid cello-oligosaccharide repeat units of MLG, the bond attacked by HTG, like that attacked by other MLG-cleaving enzymes (lichenase [67] and cellulase [68]), is likely to be precisely defined relative to the neighbouring β-(1→3) bond(s). The site of attack of lichenase on MLG is known [67], so lichenase digestion of the MXE product (whose reducing terminus is presumed to be …Glc-Glc-Glc-Glc-XXXGol) should yield a product from the reducing end of the polymer whose structure is diagnostic of the site of attack of MXE on MLG. Specifically, this would tell us the position of the closest β-(1→3) bond in the non-reducing terminal direction and therefore between which negative subsites of HTG the β-(1→3) bond sits during cleavage. Therefore, to characterise the structure of the MXE product formed with [^3^H]XXXGol as the acceptor substrate and barley MLG as the donor substrate, we digested it with lichenase. This yielded a single ^3^H-oligosaccharide (assumed to be Glc*_n_*·XXXGol), which migrated slower than [^3^H]XXXGol on TLC and was purified by preparative TLC. Graded β-d-glucosidase treatment (Supplementary Figure S1 and Mohler et al. [32]), which releases terminal β-d-glucose residues singly from an oligosaccharide, hydrolysed the Glc*_n_*·XXXGol in two steps to [^3^H]XXXGol. This indicates that the lichenase product was a nonasaccharide, Glc_2_·XXXGol. Furthermore, since there was only a single intermediate between the Glc_2_·XXXGol and XXXGol (Supplementary Figure S1), we suggest that the two β-d-Glc residues were attached to each other, as (Glc_2_)·XXXGol. In contrast, [^3^H]XXXGol with two separate Glc monosaccharide substituents attached at different positions would have given two different intermediary octasaccharides, for which there is no evidence in Supplementary Figure S1. The linkage within the (Glc_2_) disaccharide was at this point still unknown. Furthermore, the site of attachment of the (Glc_2_) moiety to the XXXGol, indicated by the dot in ‘(Glc_2_)·XXXGol’, was also still unknown.

**Figure 1.**
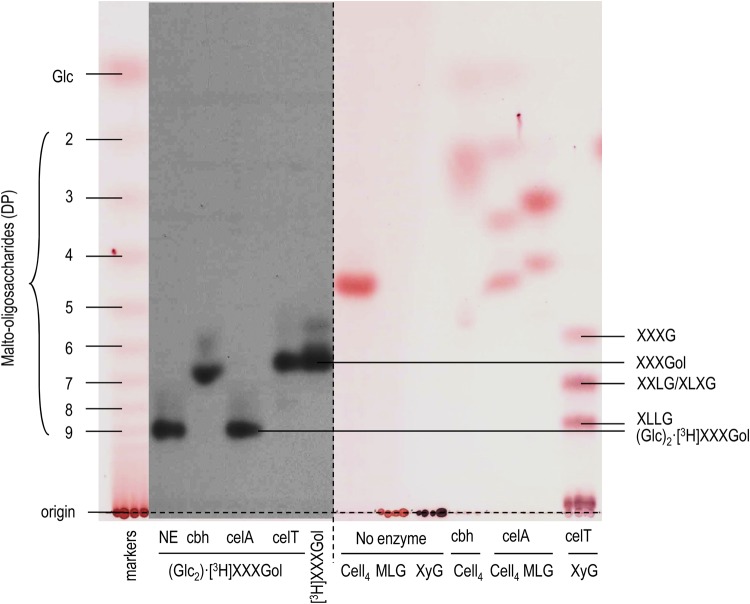
Cleavage of the glucosyl tail from (Glc_2_)·[^3^H]XXXGol by the use of three β-(1→4)-d-glucan-specific hydrolases and its proposed structure. (Glc_2_)·[^3^H]XXXGol (see Supplementary Figure S1) was incubated without enzyme (NE) or with cbh (cellobiohydrolase), celA [an *Aspergillus* β-(1→4)-d-glucanase incapable of digesting xyloglucan] or celT [a *Trichoderma* β-(1→4)-d-glucanase capable of digesting xyloglucan]. The fluorogram (centre-left) shows radioactive oligosaccharides produced from (Glc_2_)·[^3^H]XXXGol in comparison with the marker [^3^H]XXXGol. The thymol-stained control regions (left and right) show a malto-oligosaccharide ladder and three authentic β-glucose-based carbohydrates (Cell_4_, cellotetraose; MLG, barley mixed-linkage glucan; XyG, tamarind xyloglucan) before and after digestion. The TLC was developed with two ascents of the solvent.

To investigate the unknown bonds, we digested the (Glc_2_)·[^3^H]XXXGol with three hydrolases: a cellobiohydrolase and two cellulases [= endo-β-(1→4)-d-glucanases — a *Trichoderma* cellulase capable of digesting xyloglucan and an *Aspergillus* cellulase incapable of digesting xyloglucan] (Figure 1). Both the cellobiohydrolase and the *Trichoderma* cellulase were capable of digesting (Glc_2_)·[^3^H]XXXGol to [^3^H]XXXGol in the allotted time. Graded digestion of (Glc_2_)·[^3^H]XXXGol with *Trichoderma* cellulase showed that breakdown to [^3^H]XXXGol occurred without intermediate (Supplementary Figure S2), confirming that the two Glc residues are removed, and thus linked, together. *Aspergillus* cellulase did not digest the (Glc_2_)·[^3^H]XXXGol.

**Figure 2.**
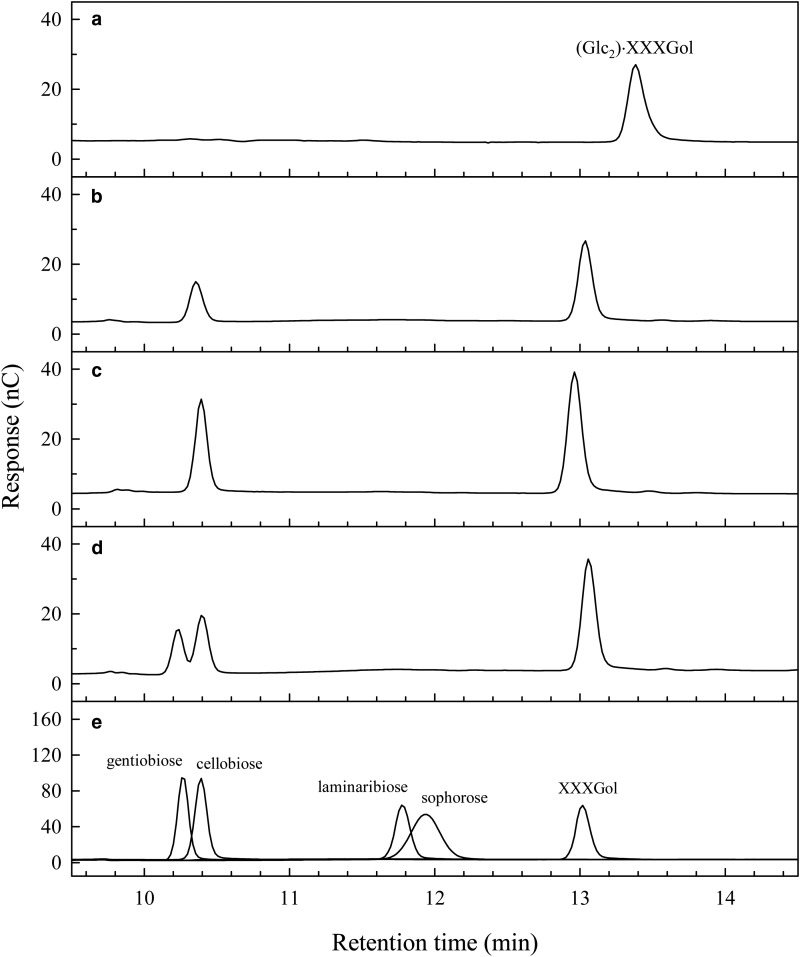
HPLC analysis of products of cbh (cellobiohydrolyase) digestion of (Glc_2_)·[^3^H]XXXGol. The (Glc_2_)·[^3^H]XXXGol was analysed alone (**a**), following cbh treatment (**b**) and following cbh treatment and subsequently spiked with cellobiose (**c**) and gentiobiose (**d**). Five markers were also separately applied (**e**). The (Glc_2_)·[^3^H]XXXGol was produced from a large batch of MXE product obtained by incubation of 2 kBq [^3^H]XXXGol in 7.5 ml of a solution containing (final concentrations) 12.5 µM non-radioactive XXXGol, 1.5% (w/v) barley MLG, 50% (v/v) *E. fluviatile* crude extract [contributing 5 mM CaCl_2_, 83 mM citrate (Na^+^, pH 6.1), 10 mM ascorbate and 1.5% w/v polyvinylpolypyrrolidone to the final reaction mixture] and 0.25% (w/v) chlorobutanol for 6 days at 25°C with constant agitation. There was 63.5% incorporation of radioactivity into 75% (v/v) ethanol-insoluble material. After lichenase digestion of this pellet, 75% (v/v) ethanol-soluble lichenase products were purified by GPC (Bio-Gel P-2), preparative TLC and preparative HPLC.

To test the nature of the bond within the (Glc_2_) disaccharide moiety, we purified (Glc_2_)·XXXGol by GPC and HPLC, then digested it with *Trichoderma* cellulase [expected to yield a non-radioactive (Glc_2_) disaccharide + [^3^H]XXXGol]. Aliquots of the digest were ‘spiked’ with authentic disaccharides and analysed by HPLC (Figure 2). Spiking allowed unambiguous discrimination between cellobiose and gentiobiose, and indicated that (Glc_2_)·XXXGol had been hydrolysed to XXXGol and cellobiose. This showed that (Glc_2_)·[^3^H]XXXGol is GG·XXXGol, where GG is cellobiose. The bond between the GG and the XXXGol moiety is also likely to be a β-(1→4)-bond because it can be cleaved by cellobiohydrolase. Such a bond identity would also be consistent with HTG's GH16 family membership.

**Figure 3.**
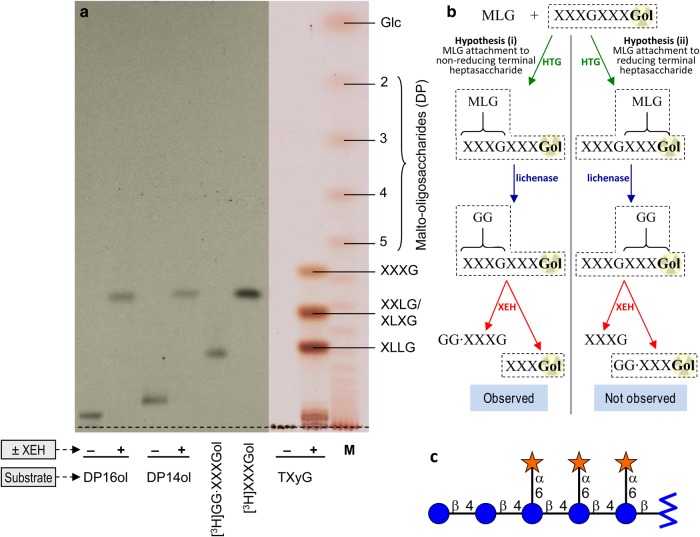
Determining which of the two subunits of XXXGXXXGol MLG is attached to during MXE activity. MLG and [^3^H]XXXGXXXGol (DP14ol) were used as donor and acceptor substrates, respectively, for the *Equisetum* MXE reaction, and the polymeric product (MLG–[^3^H]XGO-ol) was digested with lichenase. The single radioactive product [(Glc_2_)·[^3^H]XXXGXXXGol; DP16ol] was purified by GPC on Bio-Gel P-2. The DP16ol and, as a control, DP14ol were then incubated in the presence or absence of microbial XEH, and the new products were resolved by TLC (two ascents). (**a**) Portions of the plate were then fluorographed (left) or thymol-stained (right), as appropriate. (**b**) Schematic representation of the experimental strategy, indicating products that would be formed during the successive enzymic treatments according to two hypotheses for the site of attachment of MLG to the two heptasaccharide subunits of DP14ol (XXXGXXXGol, radiolabelled in the Gol moiety). Dashed boxes surround radioactive oligosaccharides. (**c**) Final deduced structure of the MXE–lichenase product, (Glc_2_)·[^3^H]XXXGol. Star, d-xylose; circle, d-glucose; zigzag, d-glucitol. Abbreviations: TXyG, tamarind xyloglucan; −/+, without and without microbial XEH digestion; M, malto-oligosaccharide marker ladder; XEH, xyloglucan endohydrolase.

As a test of whether the bond created during the MXE reaction is a β-(1→4) bond to the non-reducing terminal Glc residue of the XGO, we exploited the fact that, regardless of the length of an XGO, the only free Glc 4-hydroxy group is at the non-reducing terminus. In contrast, other free hydroxy groups also occur throughout the oligosaccharide. Therefore, if HTG attaches MLG to a Glc 4-OH, the new bond will always be to the non-reducing terminus of the XGO, whereas if it attaches it to any other hydroxy group, the new bond could be elsewhere. To test this, we used the tetradecasaccharide [^3^H]XXXGXXXGol as an acceptor substrate for MXE activity. The polymeric MXE product (MLG·[^3^H]XXXGXXXGol; washed to remove remaining free XGO) was digested with lichenase and the single radioactive product, GG·[^3^H]XXXGXXXGol (the dot indicating that we do not yet specify to which position on the [^3^H]XXXGXXXGol the cellobiose unit is attached), was purified by GPC (Bio-Gel P-2) and then digested with microbial XEH, which is expected to cleave only the single GX linkage of the [^3^H]XXXGXXXGol moiety [63]. Microbial XEH would not be expected to cleave (1→2), (1→3) or (1→6) bonds, nor bonds to xylose residues. XEH digestion of both the GG·[^3^H]XXXGXXXGol and the initial acceptor substrate ([^3^H]XXXGXXXGol) yielded a single radioactive oligosaccharide, which comigrated with [^3^H]XXXGol (Figure 3a). This indicates that MXE had attached MLG to the non-reducing terminal heptasaccharide subunit alone (Figure 3b). Since the only unique feature of the non-reducing terminal heptasaccharide is its Glc 4-OH group, this observation provides further support that the linkage formed between MLG and XXXGol by HTG is indeed β-Glc-(1→4)-β-Glc. Hence, GG·XXXGol is GGXXXGol in the standard nomenclature for xyloglucan sequences [6] (Figure 3c).

### Deducing the site of cleavage of MLG

**Figure 4.**
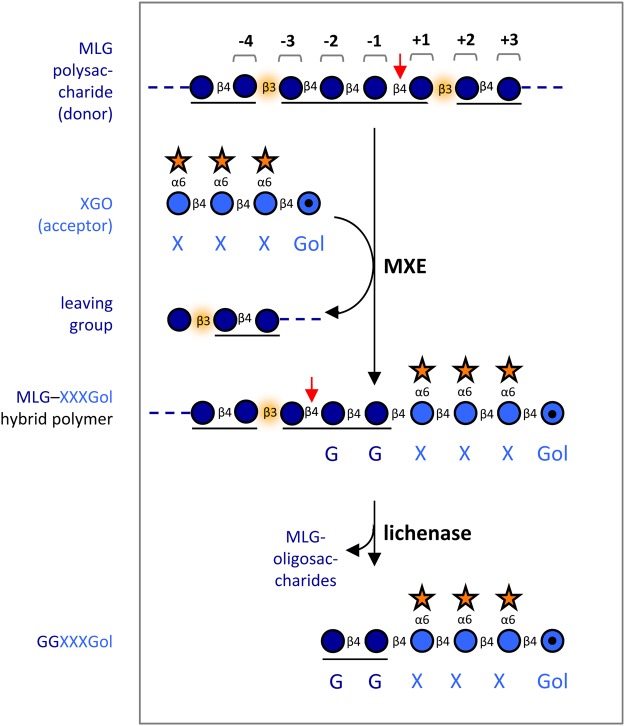
Identification of the bond initially cleaved by MXE activity. The model is based on the observation that sequential MXE:lichenase treatments yield [^3^H]GGXXXGol. Numbers with braces indicate MXE subsite identities required for MXE cleavage at the specific point; red arrows indicate sites of cleavage. Glucose residues derived from MLG and xyloglucan are coloured dark blue and light blue, respectively. Underlining indicates a cello-oligosaccharide unit. In the model, HTG cleaves a (1→4) bond within a cellotetraose unit.

Sequential MXE–lichenase treatments inform us of the position of the (1→3) bond in HTG's negative subsites during the MXE reaction. The fact that we see only a single product (GGXXXGol) means that the (1→3) bond in the negative subsites is always in the same place when the enzyme is acting on barley MLG. We reasoned that the structure of MLG substrates might affect the site of attack preferences relative to (1→3) bonds. Therefore, to further probe this apparent preference for cleavage relative to a (1→3) bond, we performed MXE–lichenase treatments on three structurally distinct MLGs: *Equisetum* MLG, barley MLG and Iceland moss MLG (known as lichenan), which have mainly cellotetraose units, mainly cellotriose units and almost only cellotriose units, respectively [64]. When each of these three MLGs were used as the HTG's donor substrate, the sole radioactive product of lichenase digestion was again [^3^H]GGXXXGol, chromatographically indistinguishable from that obtained when barley MLG was the donor (Figure 4). This confirms MXE's specificity for a (1→3) bond between subsites −4 and −3.

**Figure 5.**
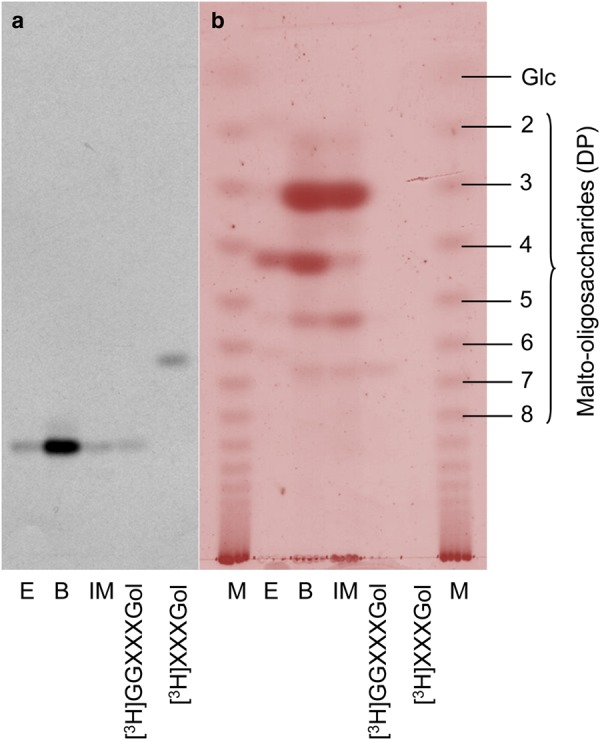
Identification of the site of cleavage of structurally dissimilar MLGs by MXE. MXE products created by incubation of reaction mixtures (20 µl) containing 15, 10 and 5 kBq [^3^H]XXXGol (84 MBq µmol^−1^) with 0.3% (w/v) *E. arvense* (E), barley (B) and Iceland moss (IM) MLG, respectively, and 50% (v/v) *E. fluviatile* crude extract for 16 h at 25°C. The 75% (v/v) ethanol-insoluble products were then digested with lichenase; lichenase products were resolved by TLC (BAW, two ascents) and detected by fluorography (**a**) and thymol staining (**b**).

If the target site of HTG is a cellotetraose unit, then the effectiveness of the three tested donor substrates should be *Equisetum* > barley > Iceland moss, since these MLGs have high, medium and low cellotetraose contents, respectively. Conversely, if the target is cellotriose, the order should be reversed. Assessed from the *V*_max_ values of MXE activity on the three tested MLGs (Supplementary Figure S2), the target is the cellotetraose unit. Given this knowledge, the deduced structure of the MXE/lichenase product, GGXXXGol (Figure 3c), and the known specificity of lichenase, we can work back to show that the site of attack of HTG on the MLG chain is within a cellotetraose unit, three bonds towards the reducing terminus from a β-(1→3) bond (Figure 5a). This suggestion assumes that HTG cleaves a β-(1→4) bond in the MLG, which would agree with the action of all other known XTH family members.

This indicates that it is a requirement for MXE activity that a (1→3) bond links the β-Glc residues occupying subsites −4 and −3, and that (1→4) bonds interlink the β-Glc residues occupying subsites −3, −2, −1 and +1.

### Does HTG prefer to cleave its donor polysaccharide near one end or near the middle?

The above experiments identify the precise molecular site (‘′’ in the sequence…G4G3G4G4G′4G3G4G…) targeted when HTG uses MLG as the donor substrate. As well as exhibiting specificity at this level, it is also possible that HTG might exhibit a preference for a general region of the MLG chain, e.g. near the non-reducing terminus, near the middle or near the reducing terminus; alternatively, the selection of G4G3G4G4G′4G3G4G sites for cleavage may be purely random with respect to the termini. Defining such preferences may help to define the biological role of HTG.

To test for any such preference, we adapted the method of Steele et al. [61]. With a size-homogeneous non-radioactive polysaccharide as the donor substrate (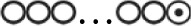) and a radioactive oligosaccharide as the acceptor (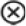), the preferred regions of cleavage can be distinguished by the size of the radioactive product —

cleavage near the non-reducing terminus:


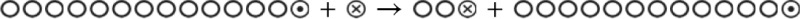


cleavage near the middle:


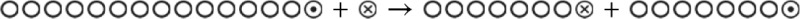


cleavage near the reducing terminus:


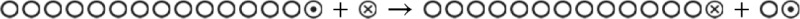


**Figure 6.**
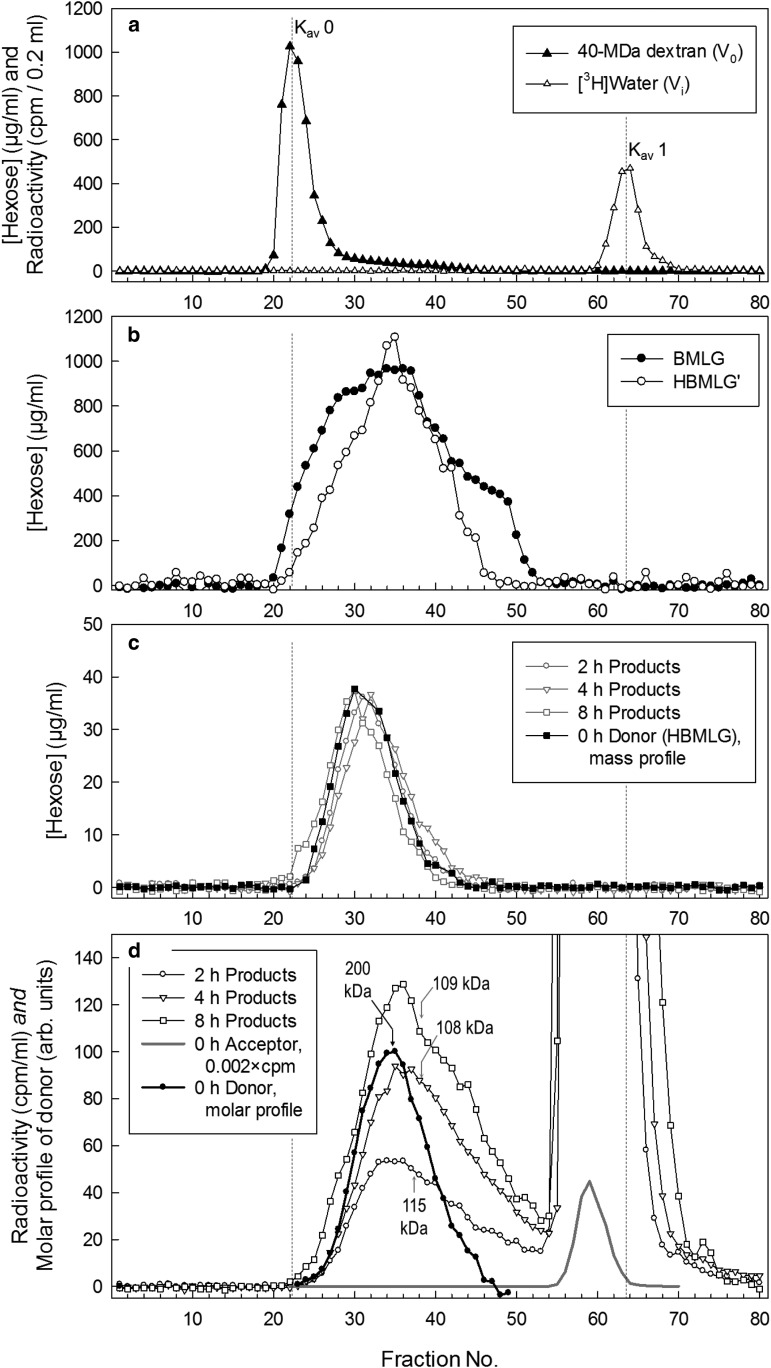
Using size-homogeneous barley MLG to explore the preferred regions of cleavage during MXE action. (**a**) Calibration of a Sepharose CL-6B column with dextran (40 MDa) and ^3^H_2_O, indicating the void volume (*K*_av_ = 0) and included volume (*K*_av_ = 1), respectively. (**b**) Elution profiles of a commercial barley MLG sample (●) and a partially size-homogeneous MLG sample (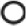). To prepare the latter, we had passed a 10-ml aliquot of barley MLG (6.25 mg/ml) through the column and pooled the fractions that had *K*_av_ ≈ 0.2–0.3 (fractions 33–37). (**c**) Three replicate preparations of partially size-homogeneous MLG were pooled and rerun through the same column; again the *K*_av_ 0.2–0.3 region was pooled as size-homogeneous MLG; final yield = 7.7% of the total starting MLG. A profile of size-homogeneous MLG is shown (▪). Aliquots of this polysaccharide were then incubated with *Equisetum* HTG in the presence of a trace of [^3^H]XXXGol for 2–8 h and rerun on Sepharose; fractions were assayed for total hexose (grey symbols), showing negligible degradation during the incubation. (**d**) Open symbols: the ^3^H profiles of the Sepharose run illustrated in (**c**), showing the radioactive transglycosylation products (MLG–[^3^H]XXXGol) and the remaining free [^3^H]XXXGol (going off-scale; the 0-h data for the oligosaccharide zone are also shown on a compressed scale; grey line). The median elution volumes of the polysaccharides in each ^3^H profile (fractions 21–53 inclusive) are shown by grey arrows (median *M*_r_ values are quoted). The ^3^H profiles show the reactants and products on a molar basis since each molecule has exactly one [^3^H]XXXGol moiety. Therefore, for comparison, we also show the molar profile of the donor substrate (●), calculated from its mass profile in (**c**, ▪) by reference to the estimated molecular mass of the MLG in each fraction [61] [using the equation *K*_av_ = 2.238–0.369(log *M*_r_)]. The black arrow (200 kDa) indicates the median of this profile.

To obtain a suitable donor substrate, we fractionated barley MLG by GPC, producing a relatively size-homogeneous polysaccharide (Figure 6a,b). We then tested this MLG as a substrate for MXE activity, re-fractionated the products at each time-point by GPC and assayed for total hexose and ^3^H. Each sample yielded a single peak of hexose residues indistinguishable from that of the initial donor substrate, indicating that only a very small proportion of the polysaccharide molecules was cleaved between 0 and 8 h — either by the MXE reaction itself or by any contaminating hydrolases (Figure 6c). In contrast, there were two radioactive peaks: unchanged oligosaccharide (acceptor substrate) centred at *K*_av_ ≈ 0.9, and a second, broader peak, attributable to the MXE reaction product (Figure 6d). The latter increased in height with increasing MXE incubation time and exhibited an elution profile which encompassed the maximum size of the donor all the way down to approximately the size of the acceptor. This indicates that MLG cleavage by MXE action does not discriminate strongly between either terminus or the middle of the MLG chain.

Hexose readings reveal the w/v concentration of MLG in solution and are not sensitive enough to detect the tracer levels of [^3^H]XXXGol; Figure 6c thus shows mass profiles of the MLG present in the solution. In contrast, the radioactive readings produce molarity profiles (Figure 6d) since each acceptor substrate molecule and each hybrid product molecule (MLG–[^3^H]XGO-ol) possess exactly one [^3^H]glucitol moiety. To allow direct comparison between the profiles of these two types of reading, we converted the mass profiles of the MLG (Figure 6c) into molar profiles (● in Figure 6d) by reference to the approximate *M*_r_ of the material in each fraction, as described previously [61]. As expected, the median *M*_r_ of the radioactive products was lower than that of the non-radioactive donor substrate when compared on this like-for-like basis (both as molar profiles). Since the donor molecules are far larger than the acceptor molecules, random cleavage should yield radioactive transglycosylation products averaging about half the *M*_r_ of the donor. Median sizes (estimated from the calibration curve of Steele et al. [61]) were estimated at 200 kDa for the donor substrate and 108–115 kDa for the ^3^H-labelled product, indicating that MXE action does indeed cleave the donor substrate essentially stochastically. In this respect, MXE appears to operate a (lack of) preference similar to that of most XTHs [61]. Our observations indicate that HTG cannot have a unique site of attack at a defined distance from either MLG terminus.

## Discussion

### Occupancy of HTG's negative subsites by MLG

Our demonstration that HTG cleaves an MLG chain three glycosidic bonds towards the reducing terminus from a (1→3) bond distinguishes this unique *Equisetum* enzyme from its GH16b subfamily co-member [69] lichenase, which cleaves MLG one glycosidic bond towards the reducing terminus from a (1→3) bond. Another difference is that lichenase requires the (1→3) bond [67], whereas β-(1→3)-linkages are clearly not a requirement for the activity of HTG since it can cleave xyloglucan and cellulose [62], which lack (1→3) bonds [2].

### Occupancy of HTG's positive subsites by MLG

Our results also give indirect information about the tolerance of (1→3) bonds in the positive subsites. Since HTG cleaves a (1→4) bond located three glycosidic bonds towards the reducing terminus from a (1→3) bond, and since MLGs contain very few cellopentaose or larger cello-oligosaccharide units, it would seem highly likely that a β-(1→3) bond usually exists between the glucose residues in subsites +1 and +2. We cannot say whether a β-(1→3) bond in this position is required for the MXE activity of HTG. To address this point, we would require polysaccharide substrates rich in cellopentaose or larger cello-oligosaccharide units. Nevertheless, the fact that no MLGOs, whether having a (1→3) or a (1→4) bond at the non-reducing end, are capable of acting as acceptor substrates — while XGOs do (Simmons et al. [62]) — suggests that HTG shows little affinity for the glucan backbone in positive subsites; the enzyme is merely capable of accommodating the continuation of MLG's backbone into the positive subsites when binding to the … G3G4G4G sequence of the donor substrate in the negative subsites. A (1→3) bond in the positive subsite is clearly *not* needed by HTG when xyloglucan or cellulose serves as the donor substrate (XET and CXE activities, respectively [62]) since these polysaccharides lack (1→3) bonds [2].

### Biological role of MXE activity and the selection by HTG of cleavage sites along the MLG polysaccharide as a whole

Extractable MXE activity (due to HTG) peaks in late-season *Equisetum* plants, whereas XET activity (due to numerous XTH isozymes resembling those of flowering plants) peaks in young, rapidly growing tissue [31,32]. It may therefore be suggested that while ‘standard’ XTHs play a role in primary cell-wall assembly and/or loosening, the MXE activity of HTG plays a strengthening role in ageing *Equisetum* stems — perhaps by linking the MLG (which extends into [18], and is sometimes restricted to [21,70], the secondary cell walls, especially those of strengthening tissues) to xyloglucan, which is predominantly in the primary wall. To fulfil a role in MLG–xyloglucan bonding most efficiently, HTG might be expected to cleave the MLG donor substrate at a site (depicted as ‘′’ below) close to the reducing terminus, so that a minimal length of the MLG chain would be lost in the form of a leaving group (depicted as 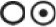) during the interpolymeric transglycosylation reaction between MLG (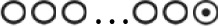, where each 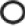 or 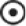 represents a cello-oligosaccharide unit) and xyloglucan (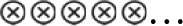, where each 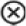 represents a cellotetraose-based unit such as XXXG), thus:


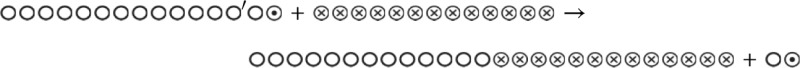


However, in contrast with this suggestion, we found that HTG attacks MLG stochastically, selecting its target cellotetraose unit randomly along the polysaccharide chain. In this respect, HTG's attack on MLG resembles XTHs' attack on xyloglucan where there is no noticeable discrimination between 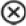 units near the reducing or non-reducing ends or near the middle of the xyloglucan chain. It thus appears that the biological function of MXE is adequately fulfilled by randomly attacking any exposed lengths of the MLG chain.

## Conclusion

In conclusion, we have defined for the first time the precise site at which HTG selects and cleaves bonds when using MLG as the donor substrate. It selects cellotetraose units, cleaving the (1→4) bond located three glycosidic bonds towards the reducing terminus from a (1→3) bond. These sites are most abundant in *Equisetum* MLG, which has a higher cellotetraose:cellotriose ratio than poalean and lichen MLG. It is unclear whether MLG or HTG appeared first during the evolution of the Equisetales; however, the enzyme is well adapted to its natural substrate. Nevertheless, an appreciable proportion of cellotetraose units do also occur in poalean MLG; thus, MXE activity might realistically be introduced into grasses or cereals by genetic manipulation. Any consequent effects on poalean wall mechanics could then provide new information on the role of HTG and potentially have the benefit of increasing wall strength in ageing stems in cereal crops. Furthermore, our data show that HTG exhibits no appreciable discrimination between cellotetraose units that are located near the reducing or non-reducing end, or middle, of MLG donor substrate chains.

## Abbreviations

BAW: butan-1-ol/acetic acid/water (2:1:1 by volume)
CBH1: cellobiohydrolase
CXE: cellulose : xyloglucan endotransglucosylase (activity)
GPC: gel-permeation chromatography
HPLC: high-pressure liquid chromatography
HTG: hetero-trans-β-glucanase (protein)
*K*_av_: elution volume on GPC relative to totally excluded polymers (*K*_av_ = 0.0) and totally included low-*M*_r_ material (^3^H_2_O; *K*_av_ = 1.0)
MLG: mixed-linkage (1→3),(1→4)-β-d-glucan
MXE: mixed-linkage glucan : xyloglucan endotransglucosylase (activity)
TLC: thin-layer chromatography
XEH: xyloglucan endohydrolase (= xyloglucan endoglucanase)
XET: xyloglucan endotransglucosylase (activity)
XGO: xyloglucan-derived oligosaccharide
XTH: xyloglucan endotransglucosylase/hydrolase (enzyme protein).
